# Amino acid supplementation of a simple inorganic salt solution supports efficient *in vitro* maturation (IVM) of bovine oocytes

**DOI:** 10.1038/s41598-019-48038-y

**Published:** 2019-08-13

**Authors:** Mohammad Bahrami, Michael B. Morris, Margot L. Day

**Affiliations:** Discipline of Physiology and Bosch Institute, School of Medical Sciences, Faculty of Medicine and Health, University of Sydney, New South Wales, Australia

**Keywords:** Cell biology, Embryology

## Abstract

Defining oocyte *in vitro* maturation (IVM) conditions allows for improved reproducibility and efficiency of bovine embryo production. IVM conditions for bovine oocytes have been extensively studied, but beneficial effects of individual supplements remain controversial. This study compared methods of cumulus oocyte complex (COC) isolation, and culture medium requirements, for IVM in order to define optimal conditions. Antral follicles in ovaries were sliced or aspirated to isolate COCs. Brilliant cresyl blue staining of COCs was used to determine the most effective collection technique and the effect of hormones and groups of amino acids in the culture medium was investigated. Our results showed COCs isolated through aspiration had greater meiotic competency to reach MII. Oocyte maturation was achieved with the addition of 1 µg/mL FSH, while estrogen and human chorionic gonadotrophin did not increase the number of MII oocytes. We also provide novel data, that supplementation of a simple inorganic salt solution with L-proline, L-glutamine and essential amino acids in combination, but not individually, resulted in nuclear maturation comparable to TCM199, a more complex medium containing all 20 common amino acids, vitamins, inorganic salts and FBS. Replacement of FBS with BSA in this simplified medium creates a defined medium which provides conditions for IVM that enable reproducible maturation rates.

## Introduction

Improving assisted reproductive technologies (ART) is beneficial to cattle production systems^1^ and efficient, reliable production of high quality bovine embryos enables genetic improvements in herds to be made, without the need to transport whole animals. However, more research is required to improve the efficiency and financial feasibility of embryo transfer in cattle^2,3^. Studying *in vitro* produced (IVP) bovine embryos allows further understanding of the culture requirements of embryos^4^, with the aim of improving developmental outcomes.

Development of IVP bovine embryos from abattoir-acquired ovaries involves the collection of cumulus oocyte complexes (COCs) from antral follicles and subsequent completion of oocyte meiotic maturation *in vitro* (IVM). Conditions for IVM of bovine oocytes have been extensively studied, but there is still no consensus regarding the optimal methods for isolation of COCs or the composition of IVM medium. Two techniques commonly used to collect COCs include slicing open the surface of follicles^5,6^ and aspiration of antral follicles^7,8^. It has been reported that slicing results in higher numbers of COCs being collected compared to aspiration^9^. Two different aspiration techniques, involving an 18 gauge needle attached to either a 5 mL syringe or a vacuum pump, have been compared to slicing, and both resulted in lower numbers of COCs collected^9^. Whilst obtaining multiple COCs from a collection may appear to be advantageous, gamete quality is critical for successful development^10,11^.

Currently the ‘best’ COCs are selected based on morphological grading^12^. Grade A COCs are those with multiple layers of clear and compact cumulus cells with a homogenous ooplasm^13^. Grade B COCs are those with at least three layers of dark compact cumulus cells and Grade C COCs have an irregularly expanded cumulus with a dark oocyte and inhomogeneous ooplasm^13^. A physiological method of selection of the most mature COCs uses brilliant cresyl blue (BCB) to determine glucose-6-phosphate dehydrogenase (G6PDH) activity within the cytoplasm of the oocyte^14^. During their growth phase, oocytes have high G6PDH activity and this enzyme degrades the BCB compound, resulting in a reduction in blue colour^15^. On the other hand, oocytes nearing the end of their growth phase have reduced G6PDH activity, and therefore have more intense blue cytoplasmic staining^14–16^. BCB staining can therefore be used to determine maturation potential of COCs isolated by different selection methods.

Supplementation of maturation medium with hormones, such as FSH, is a common practice, primarily due to the known role of FSH in recruiting follicles *in vivo*^17^. However, addition of a large range of FSH concentrations to IVM medium is reported in the literature, from 0.5–20 µg/mL, and these had varying effects on bovine COC maturation^6,18–22^. 17β-Estradiol is also commonly added to bovine COC maturation medium^6,15^. The rationale for its addition is that 17β-estradiol has a regulatory role in mammalian ovarian function^23^ and is present in follicular fluid at a concentration of 1.5 µg/mL^24^. Bovine maturation medium is often supplemented with approximately this concentration of 17β-estradiol^9,13,19^ despite reports that addition of 1 µg/mL 17β-estradiol actually reduces nuclear maturation of bovine oocytes^25^ and oocytes of other mammalian species^26^.

In addition to the variable addition of hormones, maturation medium is often supplemented with complex mixtures of amino acids^27^ without having a complete understanding of their actions. TCM199, for example, is a commercial medium that is often used for bovine IVM and contains all 20 essential and non-essential amino acids^28^ and results in high rates of nuclear maturation. In contrast, the presence of essential and non-essential amino acids in a chemically defined protein-free medium did not increase nuclear maturation of bovine oocytes^27^. Specific amino acids can have beneficial effects on oocyte maturation. For example, cysteine increases glutathione content and is important for the redox state of oocytes^29,30^, whereas other amino acids can inhibit embryo development^31^. Therefore, defining the role of individual and groups of amino acids in the *in vitro* maturation of COCs will provide insight into the metabolic requirements of oocytes.

This study aimed to compare methods of COC collection, and the use of BCB staining to determine which collection technique enables isolation of COCs that are capable of nuclear maturation. In addition, this study aimed to simplify the composition of maturation medium, by determining the requirements for FBS, exogenously added hormones and specific groups of amino acids for optimal *in vitro* maturation of bovine COCs.

## Results

### Aspiration of follicles produced oocytes with greater nuclear maturation compared to slicing open follicles

**Figure 1:**
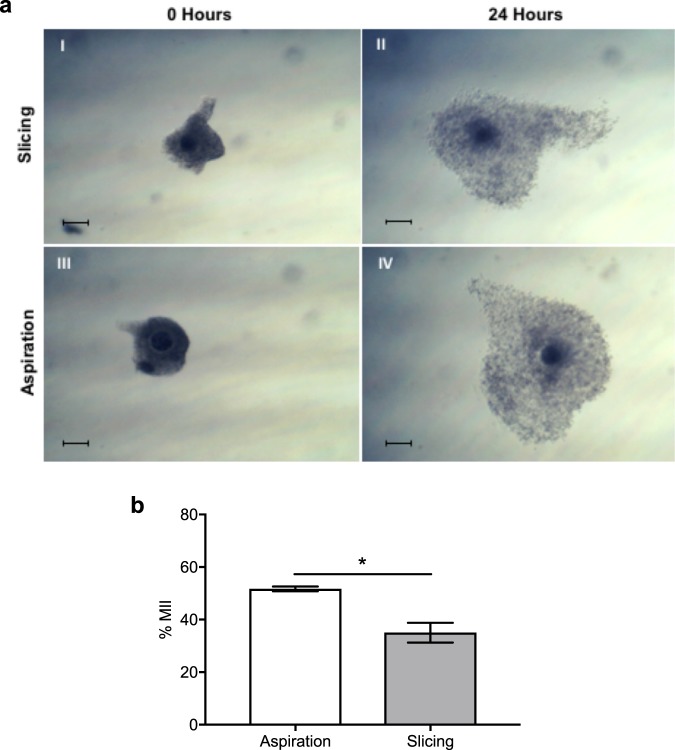
Cumulus cell expansion and nuclear maturation of bovine COCs, after 22–24 h *in vitro* maturation, following isolation by either slicing or aspiration of antral follicles. **(a)** (I) COC collected by slicing the antral follicle prior to IVM and (II) after 22–24 h IVM. (III) COC collected by aspiration of the antral follicle prior to IVM and (IV) after 22–24 h IVM. **(b)** Nuclear maturation of oocytes obtained by slicing or aspiration. Maturation of bovine oocytes was determined by the presence of a metaphase plate (MII) and an extruded polar body. *indicates *P* < 0.05 via Student’s *t*-test. Data represent mean ± SEM from 3 separate experiments with 50 to 60 COCs per treatment group.

Maturation of bovine COCs obtained by slicing open follicles on the surface of the ovary or by aspiration of antral follicles was compared to determine the technique that resulted in the higher proportion of MII oocytes. Figure 1a shows that bovine oocytes isolated from antral follicles by slicing or aspiration, and matured for 22–24 h, were capable of cumulus expansion. While the expansion of cumulus cells is reported as a marker of maturation^32,33^, we also investigated nuclear maturation using DAPI staining of oocytes and examining the presence of a metaphase plate and polar body. COCs collected by aspiration had higher (51.6 ± 0.9%) nuclear maturation compared to slicing (35.0 ± 3.8%) (n = 3, *P* < 0.05) (Fig. 1b).

**Figure 2:**
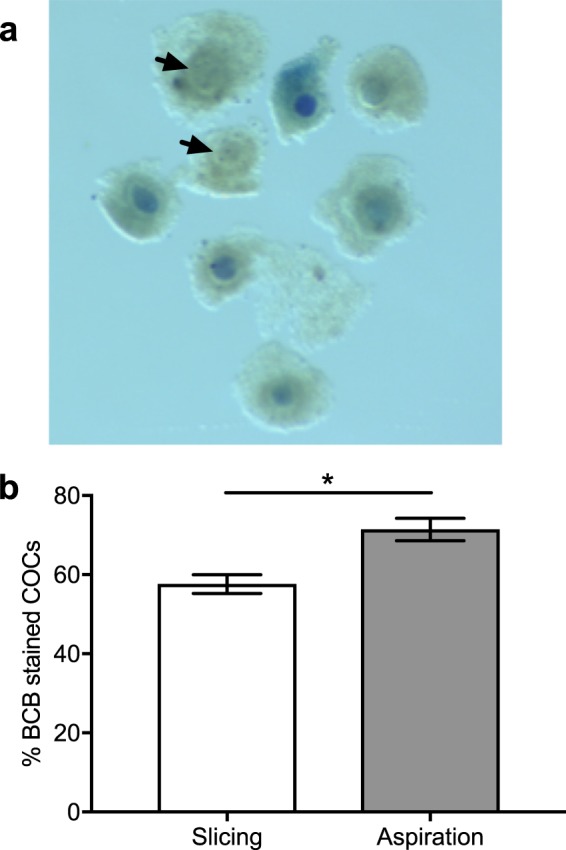
Proportion of BCB-stained COCs collected from antral follicles via aspiration and slicing. **(a)** Representative image of COCs stained with BCB. Positive staining was indicated by presence of the blue dye in the cytoplasm of the oocyte. Arrows indicate oocytes with no staining (BCB^−^). **(b)** Proportion of BCB stained COCs (mean ± SEM). *indicates *P* < 0.05 via Student’s *t*-test. Data are from 5 separate experiments, with 70 to 100 COCs per treatment group.

**Figure 3:**
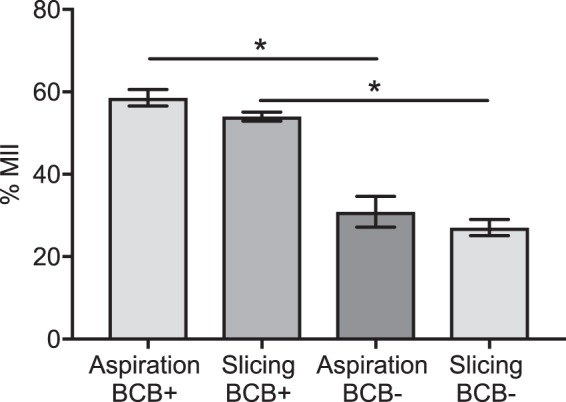
Nuclear maturation of bovine COCs subjected to BCB staining. Oocytes with positive staining of their cytoplasm (BCB^+^) and those without cytoplasmic BCB staining (BCB^−^) were matured *in vitro* for 22–24 h. Nuclear maturation of oocytes was determined by the presence of a metaphase plate (MII) and an extruded polar body. *indicates *P* < 0.05 using one-way ANOVA with Tukey’s *post-hoc* test. Data represent mean ± SEM from 5 separate experiments, with 70 to 100 COCs per treatment group.

To complement the maturation results demonstrated by DAPI staining, we stained COCs extracted by slicing or aspiration with BCB and separated the oocytes according to positive or negative staining of the cytoplasm (Fig. 2a). A higher proportion of BCB-positive (BCB^+^) oocytes (71.4 ± 2.9%) was collected by aspiration compared to slicing (57.6 ± 2.4%) (n = 5, *P* < 0.05) (Fig. 2b). Subsequent maturation of BCB^+^ COCs resulted in similar nuclear maturation for both the aspiration and slicing groups (Fig. 3). Additionally, BCB^+^ COCs had higher nuclear maturation compared to BCB negative (BCB^−^) COCs for both aspiration (58.6 ± 2.0% and 30.9 ± 3.8%, n = 5, *P* < 0.05) and slicing groups (54.0 ± 1.1 and 27.1 ± 2%, n = 5, *P* < 0.05) (Fig. 3). These results show that aspiration of bovine follicles leads to the collection of a higher percentage of oocytes that have meiotic competence.

### FSH increased nuclear maturation of bovine COCs

**Figure 4:**
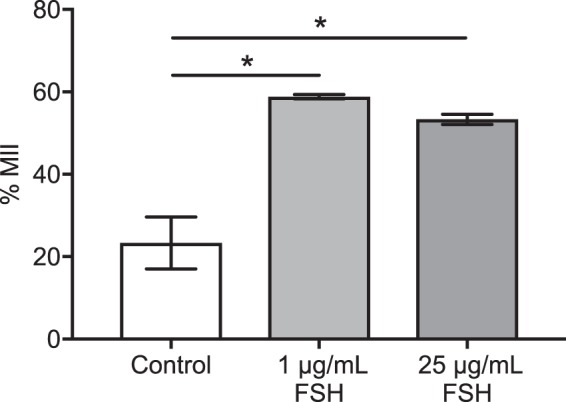
Effect of FSH on nuclear maturation of bovine oocytes *in vitro*. COCs harvested through aspiration were cultured in TCM199 without FSH (control), 1 µg/mL or 25 µg/mL FSH for 22–24 h. All treatment groups also contained 1 µg/mL 17β-estradiol. *indicates *P* < 0.05 using one-way ANOVA with Tukey’s *post-hoc* test. Data represent mean ± SEM from 3–6 separate experiments, with 20 to 50 COCs per treatment group.

The addition of 1 µg/mL FSH to TCM199 increased nuclear maturation compared to the negative control, TCM199 alone (Fig. 4). A higher concentration of FSH (25 µg/mL) did not further improve nuclear maturation. Thus 1 µg/mL FSH is sufficient for the stimulation of *in vitro* maturation of bovine oocytes.

### Addition of 17β-estradiol or hCG had no effect on nuclear maturation of bovine oocytes *in vitro*

**Figure 5:**
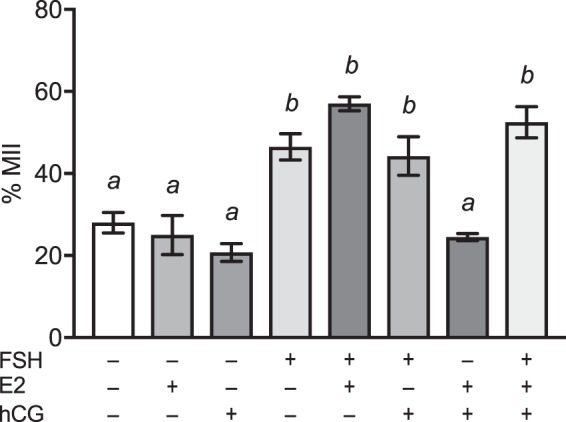
Nuclear maturation of bovine COCs in the presence of different hormone combinations. COCs harvested through aspiration were cultured in TCM199 for 22–24 h in the presence of various combinations of 1 µg/ml FSH, 1 µg/ml E2 and 0.1 IU/ml hCG. Bars with different letters are significantly different from one another, with *P* < 0.05. Analysis was conducted using one-way ANOVA with Tukey’s *post-hoc* test. Data represent mean ± SEM from 4 separate experiments, with 20 to 40 COCs per treatment group.

Maturation medium is often supplemented with other hormones such as 17β-estradiol (E2) and Luteinising hormone (LH), as these hormones are involved in follicular recruitment and maturation *in vivo*^34^. To determine whether these hormones affect nuclear maturation *in vitro*, they were added individually or in combination with 1 µg/mL FSH to TCM199 (where LH was substituted with the structurally similar human chorionic gonadotrophin (hCG)). Supplementation of TCM199 with 1 µg/mL E2 or 0.1 IU/mL hCG did not increase nuclear maturation when compared to TCM199 alone (Fig. 5). Nuclear maturation was only increased by the presence of 1 µg/mL FSH, and the addition of E2 and/or hCG along with FSH did not increase maturation further.

### Addition of amino acids to a simple inorganic salt medium supports *in vitro* nuclear maturation of bovine oocytes

TCM199 is a complex medium containing all 20 common amino acids, vitamins, and inorganic salts. We formulated a defined inorganic salt solution (M1) based on a simplified version of TCM199. M1 was then supplemented with specific amino acids to determine their effect on nuclear maturation of bovine oocytes. Additionally, a comparison of the effect of addition of BSA and FBS on nuclear maturation was performed.

**Figure 6:**
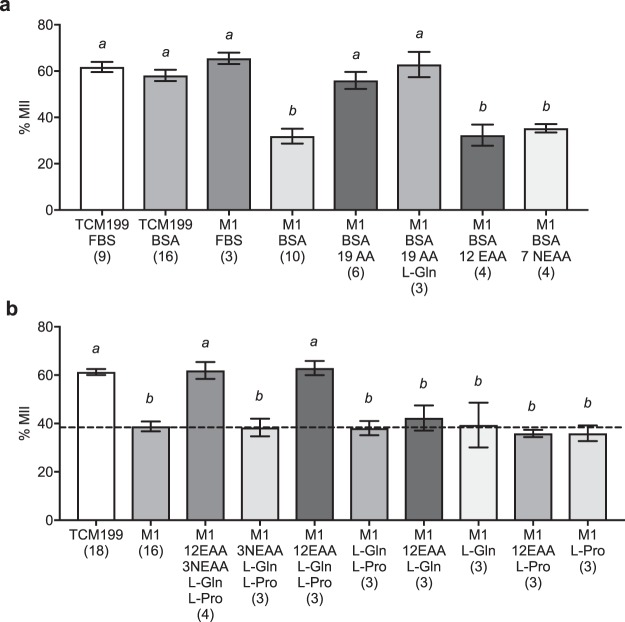
Effect of FBS, BSA and groups of amino acids on nuclear maturation of bovine COCs. (**a**) COCs, harvested by aspiration, were cultured in TCM199 or M1 for 22–24 h in the presence of either 10% FBS or 7 mg/ml BSA. M1 + 7 mg/ml BSA was supplemented with either 19 amino acids (19AA), 19AA + 2.8 mM L-Gln, 12 essential amino acids (12EAA) or 7 non-essential amino acids (7NEAA). (**b**) COCs cultured in TCM199 (+FBS) or M1 (+BSA) supplemented with groups of amino acids. Bars with different letters are significantly different from one another, with *P* < 0.05. Analysis was conducted using one-way ANOVA with Tukey’s *post-hoc* test. Data represent mean ± SEM from at least 3 separate experiments (number given in parentheses), with 30 to 40 COCs per treatment group.

There was no difference in nuclear maturation of oocytes cultured in TCM199 + 10% FBS compared to TCM199 + 7 mg/ml BSA (Fig. 6a). Furthermore, COCs matured in the inorganic salt solution M1 medium + FBS had the same nuclear maturation as TCM199, whereas nuclear maturation in M1 + BSA was significantly reduced. However, supplementation of M1 + BSA with essential and non-essential amino acids (19AA) (excluding L-Gln) resulted in nuclear maturation comparable to TCM199 + FBS or BSA and this was not further improved by inclusion of 2.8 mM L-Gln. Addition of only essential amino acids (12EAA) or non-essential amino acids (7EAA) to M1 + BSA did not improve nuclear maturation compared to M1 + BSA alone.

The effect of addition of further groupings of amino acids to M1 + BSA was examined (Fig. 6b) and showed that addition of 12EAA plus 2.8 mM L-Gln and 0.4 mM L-Pro supported the same proportion of nuclear maturation as TCM199. Whereas, addition of 12EAA and only L-Pro or L-Gln did not increase nuclear maturation.

## Discussion

In this study we provide data that will help to simplify the composition of bovine IVM medium, with the aim of obtaining improved and reproducible nuclear maturation without batch-to-batch variability. A key aim of this study was to compare two commonly used COC collection techniques, slicing and aspiration of antral follicles. It has been reported that collection by way of slicing open antral follicles does not impact nuclear maturation in bovine^9^ and or equine^35^ oocytes; rather, slicing produces a higher number of grade A COCs^36^. Our study demonstrated higher nuclear maturation of bovine oocytes using the aspiration technique compared to slicing (Fig. 1b). One possible explanation for this is that slicing may release oocytes from primordial follicles and that these oocytes could be classified as “Grade A” based solely on their multiple layers of cumulus cells and homogeneous cytoplasm^9^. However, it is likely that 22–24 h of IVM is not enough for the nuclear maturation of all these primary oocytes and our results, using a range of approaches, indicate this is the case (Figs 1b, 2 and 3).

BCB staining was used to further investigate the effect of collection techniques on nuclear maturation. BCB staining has been used to select oocytes that are likely to reach MII stage of development after 22–24 h of IVM^15^. G6PDH is abundant in growing oocytes^37^, and will break down BCB^15^, whilst oocytes further along in their development will have reduced G6PDH levels and hence stain blue (BCB^+^)^14^. Our study demonstrated that oocytes that show BCB staining at the time of isolation resulted in higher nuclear maturation (Fig. 3), as has been reported by other groups^15,16^. We also showed that aspiration of antral follicles resulted in a higher proportion of BCB^+^ oocytes in comparison to slicing (Fig. 2). This is consistent with slicing releasing primary oocytes that are in their early growth phase and would be expected to have an abundance of G6PDH. Live staining with BCB can be incorporated into a standard IVM/IVP protocol, as studies have shown higher blastocyst development when selecting oocytes using BCB^38,39^.

A critical component controlling the *in vivo* maturation of bovine oocytes is the activity of hormones, which regulate recruitment, selection and the eventual ovulation of a dominant follicle^40–43^. Thus, it is important to consider the impact of hormones on COCs during IVM. When high (25 µg/mL) and low (1 µg/mL) concentrations of FSH in the medium were compared, there was no significant difference in nuclear maturation, although nuclear maturation decreased significantly without FSH supplementation (Fig. 4). In previous studies, a range of FSH concentrations has been used^44–47^, with varied results^6,20^. The lowest concentration used in our study (1 µg/mL) is still substantially higher than the peak plasma FSH concentration (21 ng/mL) during a normal bovine estrus cycle^48^. It has been shown that 2 µg/mL FSH concentration causes abnormalities in chromosomal alignment in mice^49^ and human^50^ *in vitro* matured oocytes. Whilst our study demonstrates that 1 µg/mL FSH improves nuclear maturation, we did not investigate the effect of FSH concentration on aneuploidy.

Maturation medium used in bovine IVM protocols often include 17β–estradiol^6,15,51^. Estrogen is important in follicular recruitment *in vivo* through the suppression of GnRH release. However, in an *in vitro* system, the normal hormonal feedback loop(s) are no longer present. Studies have shown that 17β–estradiol alone reduces nuclear maturation in bovine^25^ and porcine^52,53^ oocytes. Our study confirmed that 1 µg/mL 17β–estradiol alone had no effect on nuclear maturation and does not add to the beneficial effect of FSH on IVM (Fig. 5). Based on our results, the addition of 17β–estradiol is not necessary for the *in vitro* maturation of bovine oocytes, and thus its omission helps to further simplify the composition of IVM medium.

The pre-ovulatory LH surge induces the resumption of meiosis^54^ and completion of *in vivo* follicle maturation^55^. Due to importation restrictions, it was necessary to substitute LH with the structurally similar hCG. LH and hCG act on the same receptor, luteinizing hormone-chorionic gonadotropin receptor (LHCGR)^56^. A recent study showed higher (66%) nuclear maturation in mouse GV-stage oocytes cultured in hCG in comparison to recombinant-LH (47%)^57^. In a human IVM trial, the addition of 0.5 IU/mL hCG produced comparable nuclear maturation to medium supplemented with LH^58^; however, other studies have shown that hCG does not improve nuclear maturation in patients with polycystic ovarian syndrome^59^. Our results indicate that hCG did not improve nuclear maturation of bovine COCs, regardless of whether it was added in the presence of FSH or FSH and 17β–estradiol. Future studies should compare binding of LH and hCG to the LHCGR in bovine cumulus cells and denuded oocytes to identify possible differences in downstream signalling pathways and subsequent effects on the resumption of meiosis.

This study showed that 17β–estradiol and hCG did not improve nuclear maturation when added individually or in combination with FSH (Fig. 5). Whilst others^19,51,60^ supplement maturation medium with FSH, LH and 17β–estradiol, our results show that FSH alone can support IVM of bovine oocytes (Fig. 5).

The addition of other supplements to culture medium can support the *in vitro* culture of cell lines^61^, gametes^62–64^ and embryos^60,64^. The effects of amino acids on cellular function has been investigated by a number of groups^65,66^ and supplementation of amino acids in culture medium for embryo growth and development is widely practiced^45,67^. Essential or non-essential amino acids are also added to some bovine maturation media formulations^51^, whereas premixed media, such as TCM199, contain all 20 common amino acids and are widely used for bovine IVM^6,8,25,45,60^.

Our study examined the effect of amino acids on IVM of bovine oocytes (Fig. 6). Nuclear maturation was reduced in COCs cultured in a simple inorganic salt solution, M1, in the absence of amino acids. However, nuclear maturation of COCs cultured M1 with 10% FBS was comparable to TCM199. 10% FBS contains amino acids at sufficient concentration (generally tens of micromolar) to, for example, maintain the viability of mouse ES cells^68^ and these concentrations appear to be sufficient to support nuclear maturation. Supplementation of M1 with essential and non-essential amino acids resulted in nuclear maturation comparable to TCM199 with BSA (Fig. 6), further supporting that amino acids are important constituents of maturation medium, and that their concentrations in FBS may be sufficient for maximum benefit.

This study also showed that in comparison to M1 + BSA containing both EAA and NEAA, nuclear maturation was lower in COCs when only EAA or NEAAs were present. Contrary to other reports^27^, this suggests that a combination of both essential and non-essential amino acids is necessary for the *in vitro* nuclear maturation of bovine oocytes. Furthermore, we show that a combination of EAA along with only L-Gln and L-Pro results in maturation equivalent to M1 + BSA with both EAA and all NEAAs and to TCM199 (Fig. 6b). Further experiments are needed to determine the specific amino acids required for improved bovine IVM and which, if any, are detrimental or at least superfluous. It is likely that a complex interacting network of amino acids would prove optimal, and this will include consideration of the concentrations used, rates of uptake, competition for uptake through amino-acid transporters, as well as routes of metabolism and/or use of individual amino acids in the context of the molecular mechanisms that drive maturation. These data provide a foundation for beginning this search, which can be supplemented with measuring the developmental capacity of fertilised oocytes matured in these simplified media.

## Conclusion

The purpose of this study was to compare two widely used collection techniques, as well as simplifying the culture medium used for the *in vitro* maturation of bovine oocytes. Our results suggest that aspirating antral follicles is the most effective way to collect developmentally competent COCs. Furthermore, supplementation of a simple inorganic salt solution with 7 mg/mL BSA, 1 µg/mL FSH, essential amino acids, L-Pro and L-Gln results in nuclear maturation comparable to TCM199 + FBS.

## Materials and Methods

Unless stated otherwise, all reagents were obtained from Sigma.

### Bovine ovary collection

Bovine ovaries were obtained from freshly slaughtered *Bos taurus* heifers at the local abattoir and stored in 0.9% saline +50 mg penicillin/streptomycin at 38.5 °C. All ovaries were then transported back to the laboratory, where they were washed again in pre-warmed (38.5 °C) 0.9% saline solution.

### Isolation of bovine COCs by slicing or aspiration

Bovine COCs were collected from ovarian antral follicles measuring from 2–8 mm in size. Follicles on the surface of the ovary were sliced open using an 11 mm sterile surgical blade to release intact COCs as described by others^5,6^. The ovary was then swirled in a beaker with 50 mL pre-warmed (38.5 °C) HEPES-TALP medium^69^. The contents of the beaker were transferred to a pre-warmed 50 mL tube and allowed to sediment at 38.5 °C.

Aspiration of follicles was performed using an 18-gauge needle attached to a 10 mL syringe. Follicular fluid was aspirated and transferred into a 15 mL tube where the follicles could sediment at 38.5 °C.

The resulting pellet from both groups was washed with 2 mL HEPES-TALP on a heated stage (38.5 °C) with grade A and B^70^ COCs collected and cultured at a density of 20 COCs per 100 µL drop TCM199 medium (Life Technology, Reference number: 31100–035) + 10% FBS (Life Technologies), supplemented with 1 µg/mL FSH (FoltropinV, Bioniche Life Sciences), 1 µg/mL 17β–estradiol, and 0.2 mM sodium pyruvate, unless otherwise stated.

### *In vitro* maturation and Identification of mature oocytes

COCs were cultured in a humidified incubator at 38.5 °C and 5% CO_2_ for 22–24 h. Following maturation, oocytes were stripped of cumulus cells using type IV bovine hyaluronidase (10 mg/mL) and a P200 pipette. Denuded oocytes were transferred into a microwell using a finely pulled glass pipette and fixed in 300 µL 4% paraformaldehyde in PBS for 30 min at room temperature. Oocytes were washed 3 × 300 µL PBS then permeabilised in 0.3% Triton X-100 in PBS + 1 mg/mL PVA (poly vinyl alcohol) for 20 min at room temperature. Oocytes were washed again with 3 × 300 µL PBS and transferred into 3 µL VECTASHIELD mounting medium with DAPI (Vector Laboratories H-1200) on a glass slide using a finely pulled glass pipette, and a coverslip was laid over the oocytes. Oocytes were visualised using an Olympus BX51 fluorescent microscope. Nuclear maturation was considered to have occurred based on the presence of a metaphase plate and an extruded polar body.

### Assessing oocyte developmental competence using brilliant cresyl blue

COCs harvested by slicing and aspirating were stained using BCB. After harvesting from antral follicles, washed COCs were stained with 16 µM BCB in PBS + 10% BSA (Sigma A9647, lyophilized powder) and kept at 38.5 °C in humidified air for 90 min. COCs with and without cytoplasmic staining were referred to as BCB^+^ and BCB^−^, respectively. Using a polished Pasteur pipette, COCs were washed twice in 2 mL HEPES-TALP. COCs were matured as described above for 22–24 h. Maturation was assessed by removing cumulus cells and DAPI staining the oocytes for the presence of a metaphase plate and an extruded polar body. Data was analysed using a Student’s *t*-test. The experiment was repeated 5 times.

### Effect of FSH concentration on bovine oocyte *in vitro* maturation

COCs isolated through follicle aspiration were cultured in high (25 µg/ml) and low (1 µg/ml) concentrations of FSH and matured for 22–24 h in TCM199. To assess maturation, oocytes were denuded, fixed and permeabilised as described earlier. Data was analysed through One-way ANOVA with Tukey’s *post hoc* test. All treatments were repeated 3–6 times.

### Examining the role of 17β-estradiol and hCG on bovine oocyte *in vitro* maturation

COCs isolated by follicle aspiration were matured in TCM199 supplemented 1 µg/mL FSH, 1 µg/mL 17β-estradiol and/or 0.1 IU/mL hCG for 22–24 h. To assess maturation, oocytes were denuded, fixed and permeabilised as described earlier. Data was analysed through One-way ANOVA with Tukey’s *post hoc* test. The experiment was repeated 4 times.

### The effect of amino acids on maturation of bovine COCs

Aspirated COCs were matured as described above. The culture conditions used in this experiment were TCM199 + 0.2 mM sodium pyruvate, 1 µg/mL FSH and 10% FBS or 7 mg/mL BSA; M1 medium (1.8 mM CaCl_2_, 0.81 mM MgSO_4_, 5.3 mM KCl, 26.2 mM NaHCO_3_, 117.24 mM NaCl, 1.01 mM NaH_2_PO_4_-H_2_O, 5.6 mM D-Glucose) + 0.2 mM sodium pyruvate, 1 µg/mL FSH and 10% FBS or 7 mg/mL BSA.

Additionally, 50x MEM (ThermoFisher #11130051) and 100x MEM NEAA (ThermoFisher #11140076) were diluted in M1 to generate treatment groups that included 12 essential amino acids (12EAA) (final concentrations: 0.419 mM L-Arg, 0.07 mM L-Cystine (L-Cys), 0.14 mM L-His, 0.28 mM L-Ile, 0.28 mM L-Leu, 0.277 mM L-Lys, 0.07 mM L-Met, 0.14 mM L-Phe, 0.28 mM L-Thr, 0.035 mM L-Trp, 0.139 mM L-Tyr, 0.28 mM L-Val) and 7 non-essential amino acids (7NEAA) (final concentrations: 0.3 mM Gly, 0.3 mM L-Ala, 0.3 mM L-Asn, 0.3 mM L-Asp, 0.3 mM L-Glu, 0.3 mM L-Pro and 0.3 mM L-Ser). A combination of both essential and non-essential amino acids (19AA) with 2.8 mM L-Glutamine (19AA + L-Gln) was also formulated. A mixture of 3 NEAA was also used containing 0.66 mM Gly, 0.28 mM L-Ala and 0.24 mM L-Ser. L-Pro and L-Gln were used alone at a concentration of 0.4 mM and 2.8 mM, respectively. To assess maturation, oocytes were denuded, fixed and permeabilised as described earlier. Data was analysed through One-way ANOVA with Tukey’s *post hoc* test. All treatments were performed at least three times.

### Statistical analysis

Data are presented as mean ± standard error (SEM). All experiments were repeated at least 3 times, with a minimum of 20 COCs per treatment group in each experiment. Statistical analysis was performed using GraphPad Prism v7. Statistical tests used are described in the relevant methods section.

## Data Availability

All data generated or analysed during this study are included in this manuscript.
